# Interpretation von EPRD-Daten im Kontext der EndoCert-Zertifizierung

**DOI:** 10.1007/s00132-026-04820-1

**Published:** 2026-04-15

**Authors:** Katrin Osmanski-Zenk, Oliver Melsheimer

**Affiliations:** 1 Orthopädische Klinik und Poliklinik, Universitätsmedizin Rostock, Doberaner Straße 142, 18057 Rostock, Deutschland; 2 EPRD Deutsche Endoprothesenregister gGmbH, Berlin, Deutschland

**Keywords:** Endoprothetik, Qualitätssicherung im Gesundheitswesen, Endoprothetikzentrum, Ausfallwahrscheinlichkeiten, Ergebnisqualität, Arthroplasty, Health care quality assurance, Certified Arthroplasty Center, Prosthesis failure, Treatment outcome

## Abstract

**Hintergrund:**

Die externe Qualitätssicherung in der Endoprothetik in Deutschland stützt sich maßgeblich auf das EndoCert-Zertifizierungssystem und das Endoprothesenregister Deutschland (EPRD). Beide Systeme liefern umfangreiche Daten, deren Mehrwert sich jedoch erst durch eine methodisch korrekte und kontextualisierte Interpretation erschließt.

**Ergebnisse:**

Ziel dieser Arbeit ist es, darzustellen, welche EPRD-Daten im Kontext der EndoCert-Zertifizierung relevant sind und wie diese qualitätsorientiert analysiert und interpretiert werden können. Es werden die zentralen Datenquellen des EPRD, wie Jahresübersicht, Klinikauswertung, PROM-Berichte und Jahresbericht, systematisch eingeordnet und deren Aussagekraft im Hinblick auf EndoCert-Anforderungen erläutert.

**Schlussfolgerung:**

Die strukturierte Nutzung und Interpretation der EPRD-Daten ermöglicht EndoCert-Zentren eine differenzierte Bewertung ihrer Ergebnisqualität und unterstützt eine datenbasierte Qualitätssteuerung im Zertifizierungsprozess.

## Abstract (en)

**Background:**

External quality assurance in arthroplasties in Germany is largely based on the EndoCert certification system and the German Arthroplasty Registry (EPRD). Both systems provide extensive data; however, their added value emerges only through methodologically sound and contextualized interpretation.

**Results:**

This article aims to outline which EPRD data are relevant in the context of EndoCert certification and how these data can be analyzed and interpreted in a quality-oriented manner. The key EPRD data sources, like annual overview, hospital-specific reports, PROM reports, and the annual report, are systematically categorized, and their informative value is discussed with regard to EndoCert requirements.

**Conclusion:**

Structured use and interpretation of EPRD data enable EndoCert centers to perform a differentiated assessment of outcome quality and support data-driven quality management within the certification process.

Die EndoCert-Zertifizierung stützt sich zunehmend auf registerbasierte Ergebniskennzahlen aus dem Endoprothesenregister Deutschland (EPRD). Der Beitrag zeigt, welche EPRD-Daten für zertifizierte Endoprothetikzentren relevant sind und wie diese interpretiert werden sollten, um ihre Ergebnisqualität valide zu bewerten und gezielt in die klinische Qualitätssteuerung zu überführen.

## Hintergrund

EndoCert ist ein zertifizierungsbasiertes Qualitätssicherungssystem für Endoprothetikzentren (EPZ) und Endoprothetikzentren der Maximalversorgung (EPZmax). Es bewertet Kliniken anhand von Struktur‑, Prozess- und Ergebnisparametern. Zu den wesentlichen Strukturvoraussetzungen zählen u. a. Mindestmengen (pro Standort und Operateur), Qualifikation der Operateure (Hauptoperateure, Senior-Hauptoperateure, spezieller orthopädischer Chirurg) und auch interdisziplinäre Kooperationen. Während sich die Prozessqualität durch standardisierte Behandlungspfade, festgelegte interdisziplinäre Besprechungsroutinen und klar definierte Stellen- und Schnittstellenbeschreibungen auszeichnet, umfasst sie zudem die strukturierte Indikationsstellung sowie die prä- und postoperative radiologische Diagnostik einschließlich der systematischen Beurteilung der Implantatposition und -funktion. Da sich die im Rahmen der EndoCert-Zertifizierung erfasste Ergebnisqualität im stationären Setting primär auf klassische Qualitätsindikatoren wie beispielsweise postoperative Infektionen, Re-Interventionen oder neurologische Komplikationen und radiologische Parameter beschränkt, verpflichtet EndoCert die zertifizierten Einrichtungen seit Beginn zur Teilnahme am Endoprothesenregister Deutschland (EPRD) [6]. Durch die verpflichtende Meldung aller endoprothetischen Eingriffe an das Register wird es möglich, die Ergebnisqualität auch langfristig abzubilden. Insbesondere die im EPRD ermittelten Ausfallwahrscheinlichkeiten und Implantatstandzeiten ermöglichen eine über die reine Krankenhausverweildauer hinausgehende Bewertung der Ergebnisqualität und stellen damit eine essenzielle Ergänzung zu den EndoCert-Qualitätsparametern dar. Seit 2025 werden die im EPRD generierten Berichte nicht mehr nur als ergänzende Informationsquelle herangezogen, sondern sind als klar definierte und prüfbare Kennzahlen in den Zertifizierungsprozess von EndoCert eingeflossen. Damit wurden sowohl die Vollständigkeit der Falldokumentation, einschließlich der jährlichen Meldequote, als auch zentrale Ergebnisparameter der endoprothetischen Versorgung zu verbindlichen Bewertungsgrößen. Die Fachexperten und letztlich der Zertifikatserteilungsausschuss berücksichtigen seither die registerbasierten Auswertungen systematisch bei der Bewertung der Ergebnisqualität, hinsichtlich langfristiger Standzeiten, revisionsbezogener Kennzahlen und Auffälligkeiten im Versorgungsverlauf. Die Berücksichtigung der EPRD-Daten erfolgt dabei vorrangig durch die und in der zu zertifizierenden Einrichtung selbst. Eine Einsichtnahme des EPRD-Klinikberichts findet nur im Audit durch den Fachexperten statt, der auf dieser Grundlage die von der Klinik abgegebene Analyse der EPRD-Ergebnisse und die daraus abgeleiteten Maßnahmen bewertet. Damit erfährt die Nutzung der EPRD-Daten innerhalb des EndoCert-Systems eine deutliche strukturelle und methodische Aufwertung, da die bislang eher heterogen berücksichtigten Registerergebnisse auf Klinikebene nun durch klar definierte und prüfbare Kennzahlen einer systematischen Bewertung zugänglich gemacht werden.

Insgesamt lieferten im Jahr 2024 721 Kliniken Daten an das EPRD, davon waren 497 Einrichtungen in Deutschland nach EndoCert zertifiziert, was einem Anteil von rund 69 % entspricht. Im selben Datenjahr wurden 410.333 Dokumentationen im Register erfasst [2]. Entsprechend den EndoCert-Anforderungen, nach denen alle relevanten endoprothetischen Eingriffe an das EPRD gemeldet werden müssen, stellen EndoCert-Eingriffe den überwiegenden Teil der im EPRD dokumentierten Registerfälle dar.

## Bedeutung einer präzisen Interpretation der EPRD-Daten im Kontext der EndoCertZertifizierung

Der EPRD-Jahresbericht 2025 veröffentlichte, dass sich die Ausfallwahrscheinlichkeiten in der Hüftendoprothetik seit Etablierung des Registers über die Zeit nicht substanziell reduziert haben. Im Gegensatz dazu ist in der Knieendoprothetik eine Verbesserung der Ergebnisse erkennbar [2].

Die strukturierte und zentrenspezifische Auswertung der EPRD-Daten identifiziert Optimierungspotenziale in den Endoprothetikzentren

Vor diesem Hintergrund kommt der zentrenspezifischen Analyse der EPRD-Daten im Rahmen der EndoCert-Zertifizierung eine zentrale Funktion zu, da sie maßgeblich dazu beiträgt, Risikoentwicklungen auf Klinikebene frühzeitig zu erkennen und qualitätsrelevante Steuerungsimpulse abzuleiten. Speziell für die Hüftendoprothetik wird deutlich, dass das bloße Vorliegen registerbasierter Kennzahlen nicht ausreicht, um die Versorgungsqualität nachhaltig zu verbessern. Erst die qualifizierte Interpretation der EPRD-Daten, differenziert nach z. B. Implantattyp, Patientenkollektiv, Risikoprofil und Einrichtungsstrukturen, ermöglicht es EndoCert-Zentren, potenzielle Qualitätsdefizite zu identifizieren und Verbesserungsmaßnahmen abzuleiten. Angesichts des hohen Anteils der EndoCert-Kliniken im EPRD werden EPZ/EPZmax durch eine systematische, vollumfängliche und nachvollziehbare Registerdatenanalyse nicht nur zur Optimierung ihrer eigenen Ergebnisqualität beisteuern, sondern leisten zugleich einen wesentlichen Beitrag zur Verbesserung der bundesweiten endoprothetischen Versorgung. In einer früheren Untersuchung konnte unsere Arbeitsgruppe zeigen, dass regelmäßige, strukturierte Rückmeldungen im Rahmen der EPRD-Klinikauswertungen mit einer Verbesserung beziehungsweise Stabilisierung der Versorgungsqualität in der Hüft- und Knieendoprothetik assoziiert sind [11]. International zeigen Registerdaten, dass sich die Revisions- bzw. Ausfallwahrscheinlichkeiten sowohl in der Hüft- als auch Knieendoprothetik über die Zeit messbar verbessert haben [1, 9, 12]. Vor diesem Hintergrund eröffnet die strukturierte und zentrenspezifische Auswertung der EPRD-Daten durch EndoCert-Zentren die Möglichkeit, gezielt Optimierungspotenziale zu identifizieren und dadurch langfristig die Hüftrevisionsraten auch in Deutschland zu senken und zugleich die Ergebnisqualität der endoprothetischen Versorgung insgesamt weiter zu verbessern. Eine konsequente Nutzung dieser Daten im Rahmen der EndoCert-Zertifizierung ist daher essenziell, um die Versorgungsqualität national nachhaltig zu steigern.

## Relevante EPRD-Daten im Kontext der EndoCert-Zertifizierung

Das EPRD stellt den EPZ/EPZmax eine Vielzahl an Daten bereit, die für die Bewertung der Ergebnisqualität im Rahmen der EndoCert-Zertifizierung von Bedeutung sind. Dazu zählen klinikbasierte Jahresübersichten zu Versorgungsformen und Patientencharakteristika, Informationen zu eingesetzten Verfahren sowie die Meldequote, die als zentraler Qualitätsparameter gilt (Tab. 1). Darüber hinaus liefern die Klinikauswertungen differenzierte Angaben zu Ausfallwahrscheinlichkeiten und Mortalitätsraten. Ergänzend zur Klinikauswertung stellt das EPRD den Zentren eine fallbezogene Excel-Auswertung zu den aufgetretenen Folgeeingriffen zur Verfügung. Seit 2025 wurden technische Schnittstellen etabliert, über die alle zertifizierten EndoCert-Zentren patientenberichtete Ergebnismessungen (PROM), sofern diese im Register hinterlegt sind, für eine vertiefte Ergebnisanalyse heranziehen können.

**Tab. 1:** Übersicht klinikspezifischer Kennzahlen aus der EPRD-Jahresübersicht, deren Bedeutung für die Zertifizierung und Interpretationsmöglichkeiten. Parameter mit einem Sternchen (*) können zusätzlich mit den Daten aller EPRD-Kliniken verglichen werden.

Parameter/Kennzahl	Bedeutung für EndoCert	Interpretationsmöglichkeiten
Anzahl aller gemeldeten Eingriffe	Grundlage für die Berechnung der zentrumsindividuellen Meldequote	Hohe Meldequote gewährleistet die Validität und Aussagekraft aller Auswertungen im EPRD und stellt sicher, dass registerbasierte Qualitätsbewertungen für ein Zentrum belastbar und auditrelevant genutzt werden können
	Einer der zentralen Qualitätsindikator innerhalb des EndoCert-Systems	Geringe Meldequote weist auf potenzielle Erfassungsdefizite hin, die die Aussagekraft aller Analysen einschränken und im Zertifizierungsprozess kritisch zu bewerten sind
Anzahl aller Eingriffe je Versorgung (Hüfte vs. Knie, elektiv vs. Wechsel, totaler Femurersatz)*	Grundlage zur Bewertung der Mindestmengen, des Versorgungsspektrums und der Einhaltung endoprothetischer Schwerpunktsetzungen	Ermittelt Schwerpunktbildungen und ermöglicht Vergleichbarkeit der eigenen Leistungsstruktur mit bundesweiten Profilen
Auswertbarkeit aller Eingriffe*	Zentraler Qualitätsindikator der Dokumentationsqualität, die Voraussetzung für auditrelevante Registerauswertungen ist	Niedrige Auswertbarkeit kann auf strukturelle oder technische Mängel im Meldeprozess hinweisen und gefährdet die Aussagekraft der Qualitätsbewertung
Anzahl aller Weiterbildungseingriffe*	Relevant für die Bewertung der Weiterbildungsbefugnis sowie der kontrollierten Einbindung von Weiterzubildenden in die Versorgung	Erlaubt Einschätzungen zur operativen Weiterbildungsstruktur und potenziellen Auswirkungen auf Ergebnisqualität
		Erhöhte Anteile solcher Eingriffe können bei gleichzeitig stabilen Ergebnisqualitäten auf eine gut strukturierte Weiterbildungsorganisation hindeuten
		Geringe Anteile oder Verschlechterungen der Ergebnisse geben Hinweise auf unzureichend etablierte Weiterbildungsstrukturen
Anzahl aller Eingriffe unter EndoCert-Bedingungen*	Kernindikator, da nur diese Fälle in den zertifizierungsrelevanten EPRD-Auswertungen berücksichtigt werden	Erlaubt die Abgrenzung der qualitätsgesicherten Versorgung
	Korrekte Fallzuordnung ist zentral für die Aussagekraft der EndoCert-bezogenen Klinikauswertung	Je größer der Anteil, desto zuverlässiger lassen sich zentrenspezifische Qualitätsniveaus, Implantatstrategien und Ergebnisverläufe abbilden
Anzahl verwendeter Implantatsysteme	Relevant für die Beurteilung von Standardisierung, Material- und Systemkontinuitäten sowie der Einhaltung zertifizierter Implantatportfolios	Hohe Systemvielfalt kann auf fehlende Standardisierung, potenziell variierende Ergebnisqualität und auf spezialisierte Versorgungsanforderungen hindeuten
	Grundlage zur Überprüfung, ob für alle verwendeten Systeme gültige Schulungsnachweise der Operateure und des Operationsteams vorliegen	Eine begrenzte, funktional differenzierte Systemvielfalt im Sinne eines stringenten Implantatportfolios (z. B. Standardschaft, Kurzschaft, Konussystem innerhalb klar definierter Konzepte) kann auf strukturierte Versorgungsstrategien, klare Indikationspfade und gezielte Schulung des Operationsteams hindeuten
Übersicht Patientencharakteristika (Alter, Geschlecht, BMI) je Versorgung*	Grundlage zur Interpretation risikoadjustierter Qualitätskennzahlen	Abweichungen vom bundesweiten Patientenprofil können auf eine besondere Versorgungsschwerpunktsetzung oder ein erhöhtes Risikoprofil des Zentrums hindeuten
	Bildet die Fallkomplexität und Risikostruktur eines Zentrums ab	Diese sind bei der Interpretation von Ausfallwahrscheinlichkeiten, Komplikationsraten etc. zwingend zu berücksichtigen
Übersicht Voroperationen je Versorgung*	Wichtiger Risikofaktor, da die Häufigkeit und Art der Voroperationen die Risikostruktur des Zentrums widerspiegeln	Hohe Voroperationsraten deuten auf komplexere Versorgungsprofile hin und sind essenziell zur Beurteilung von Ausfallwahrscheinlichkeiten
	Wesentliche Grundlage für die fallbezogene Risikocharakterisierung im Zertifizierungskontext	
Übersicht Frakturversorgung je Versorgung*	Relevante Abgrenzung gegenüber elektiven Eingriffen, da Frakturen substanzielle ergebnisrelevante Risikofaktoren darstellen	Erklärt Unterschiede im Risikoprofil und ermöglicht die bessere Einordnung zentrumspezifischer Revisions- oder Mortalitätsraten
		Erklärt ggf., warum ein Teil der Eingriffe nicht unter EndoCert-Bedingungen erfolgte
Übersicht Versorgungsformen je Versorgung*	Charakterisierung des operativen Spektrums und seiner Komplexität im Zertifizierungskontext	Unterschiede in den Versorgungsformen können zur Erklärung unterschiedlicher Outcome-Profile herangezogen werden
Übersicht Verankerungstechniken je Versorgung*	Prüfung, ob die gewählten Fixationsstrategien den EndoCert-Vorgaben zur indikationsgerechten Versorgung und den zugrunde liegenden Behandlungskonzepten des Zentrums entsprechen	Unterschiede in den Verankerungsstrategien lassen sich im Kontext der Patientencharakteristika, z. B. des Alters, sinnvoll bewerten und liefern Hinweise auf die Anpassung der Implantatwahl an patientenspezifische Risiken
Übersicht verwendete Implantattypen (+ Größen, + Material) je Versorgung*	Bewertung der Standardisierung und Kontinuität des Implantatportfolios	Abweichungen vom bundesweiten Einsatzspektrum können auf bewusst gewählte Schwerpunktsetzungen oder auf strukturelle Besonderheiten des Zentrums hinweisen
		Es lässt sich prüfen, ob selten verwendete Implantattypen Rückschlüsse auf Ergebnisqualität oder Implantatperformance zulassen
Wechselgründe*	Zentral für die Bewertung typischer Versagensmuster	Erlaubt die Identifikation häufiger Revisionsanlässe, unterstützt die Ableitung zentrenspezifischer, aber auch zentrumsübergreifender Verbesserungsansätze (z. B. Optimierung der Infektionsprävention, der Implantatstrategie oder der Nachbehandlung)
	Die systematische Erfassung der Revisionsgründe ermöglicht es EPZ/EPZmax, ihre Behandlungsstrategien und Implantatauswahl auf häufige Problemfelder auszurichten	Bei gehäuften Zuverlegungen können zudem Kooperationsstrukturen mit umliegenden EPZ bzw. EPZmax überprüft und gezielt weiterentwickelt werden
Übersicht der gewechselten Komponenten*	Ergänzt die Ursachenanalyse, ermöglicht die Bewertung komponentenspezifischer Schwachstellen oder Versorgungsstrategien *(wobei darauf zu achten ist, dass der Patient nicht zwingend vorher in dem EPZ/EPZmax versorgt worden sein muss …)*	Erlaubt Identifikation von Mustern, z. B. gehäufte Wechsel bestimmter Komponenten, die auf implantatspezifische Problematiken oder operative Technikvariationen hinweisen

*BMI* Body-Mass-Index,* EPRD* Endoprothesenregister Deutschland, *EPZ/EPZmax* Endoprothetikzentrum/Endoprothetikzentrum der Maximalversorgung

Damit stehen im Kontext der Zertifizierung sowohl klassische harte Endpunkte wie Revisionsereignisse als auch potenziell patientenzentrierte Outcome-Daten für eine strukturierte Interpretation zur Verfügung.

Für eine übergeordnete Einordnung der eigenen Ergebnisse ist zudem der jährliche EPRD-Registerbericht heranzuziehen. Auch wenn dieser keine zentrenspezifische Auswertung darstellt, liefert er wichtige Kontextinformationen auf Implantat- und Versorgungsebene, einschließlich der Darstellung von Ausfallwahrscheinlichkeiten sowie zentraler Einflussfaktoren wie Alter, Geschlecht oder Klinikgröße.

Der zentrale Mehrwert der EPRD-Daten im EndoCert-System liegt nicht allein in der Datenerhebung, sondern in deren strukturierter Interpretation und Nutzung innerhalb etablierter Qualitäts- und Risikomanagementprozesse der zertifizierten Zentren.

### Jahresübersicht

Die EPRD-Jahresübersicht stellt klinikspezifische Basiskennzahlen zur Versorgungsrealität bereit (Tab. 1) und bildet damit einen zentralen Ausgangspunkt für die strukturierte Interpretation der Ergebnisqualität im Kontext der EndoCert-Zertifizierung.

**Abb. 1:**
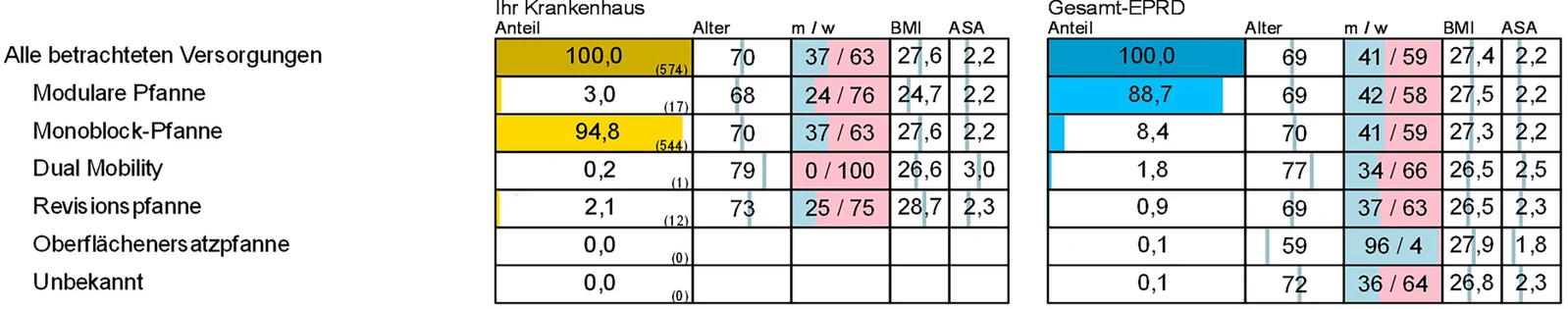
Auszug aus einer EPRD (Endoprothesenregister Deutschland)-Jahresübersicht am Beispiel der Pfannentypverteilung bei elektiver Hüftendoprothetik. *BMI* Body-Mass-Index, *ASA* American Society of Anesthesiologists, *m* männlich, *w* weiblich

Die Abb. 1 zeigt exemplarisch die Möglichkeiten der EPRD-Jahresübersicht zur Einordnung zentrenspezifischer Versorgungsstrategien im Vergleich zur bundesweiten Versorgung. Dargestellt ist der Anteil unterschiedlicher Pfannentypen bei elektiver Hüftendoprothetik einer Klinik im Vergleich zu allen am EPRD teilnehmenden Kliniken. Während im dargestellten Zentrum Monoblockpfannen in 94,8 % der Fälle eingesetzt werden, liegt ihr Anteil im bundesweiten Vergleich bei lediglich 8,4 %. Solche Abweichungen sind nicht per se als Qualitätsmerkmal zu interpretieren, liefern jedoch einen wichtigen Kontext für die Bewertung von Ergebniskennzahlen und unterstreichen die Notwendigkeit einer differenzierten, zentrenspezifischen Interpretation.

### Klinikauswertung

Die Klinikauswertung des EPRD stellt das zentrale Instrument zur zentrenspezifischen Analyse der Ergebnisqualität dar. Für die EndoCert-Zertifizierung ist sie in zweifacher Hinsicht relevant: Zum einen ermöglicht sie eine differenzierte Abbildung der Versorgungsrealität eines Zentrums, einschließlich Fallzahlen, Risikoprofil, Implantatportfolio und Ausfallwahrscheinlichkeiten. Zum anderen erlaubt sie den direkten Vergleich dieser Kennzahlen mit allen am EPRD teilnehmenden Kliniken und schafft damit eine externe Referenzbasis für die Bewertung der eigenen Ergebnisqualität [5]. Ergänzend stellt das EPRD den Zentren eine fallbezogene Auswertung in Form einer Excel-Datei zur Verfügung, die, bei vorliegender Patienteneinwilligung, detaillierte Informationen zu den im jeweiligen Haus durchgeführten Versorgungen, die im Betrachtungszeitraum bereits im selben oder einem anderen Haus erneut operiert werden mussten, beinhaltet und eine vertiefte, einzelfallbezogene Analyse auffälliger Verläufe ermöglicht. Diese enthält neben dem Zeitpunkt und der Art des Folgeeingriffs auch Angaben zu den dokumentierten Revisionsursachen. Dabei kommt insbesondere infektionsassoziierten Folgeeingriffen, gekennzeichnet durch den Diagnosecode T84.5, eine besondere Bedeutung zu, da sie als qualitätsrelevante Endpunkte sowohl im Registerkontext als auch im Rahmen der EndoCert-Zertifizierung bewertet werden.

#### Relevante EndoCert-Anforderungen zur Ergebnisqualität

EndoCert fordert ausdrücklich die systematische Erhebung klinikspezifischer Ergebniskennzahlen, insbesondere zu Implantatstandzeiten, Komplikationen, Folgeeingriffen und Ausfallwahrscheinlichkeiten. Diese Kennzahlen sollen nicht nur dokumentiert, sondern aktiv im Rahmen des Qualitäts- und Risikomanagements genutzt und nachvollziehbar interpretiert werden. EndoCert unterscheidet zwischen Primär- und Folgeeingriffen der Hüft- und Knieendoprothetik unter Einschluss elektiver sowie frakturbedingter Versorgungen. Die Einbindung externer Qualitätssicherungssysteme, wie das EPRD, ist explizit Bestandteil der EndoCert-Anforderungen [7].

#### Neuerung in der Klinikauswertung

Mit der aktuellen Klinikauswertung, die Mitte Dezember 2025 den teilnehmenden Kliniken zur Verfügung gestellt wurde, erfolgte eine inhaltliche Neuausrichtung mit einem verstärkten Fokus auf die Darstellung kurzfristiger Ergebnisse in der primären Hüft- und Knieendoprothetik sowie mittelfristiger Ergebniskennzahlen. Diese Weiterentwicklung ist im Zusammenhang mit der EndoCert-Zertifizierung von besonderer Bedeutung, da kurzfristige Veränderungen der Ausfallwahrscheinlichkeiten nun differenzierter analysiert werden können. Die Auswertungen erfolgen getrennt nach elektiver Hüftendoprothetik mit zementfreiem bzw. zementiertem Schaft, Hemiendoprothesen sowie nach nichtelektiven Hüftversorgungen und nach Standard-Knie-TEP, Constrained-Knie-TEP, Unischlitten und letztlich femoropatellaren Knieversorgungen. Ergänzend werden risikoadjustierte kurzfristige Ergebnisse ausgewiesen, wodurch auch Zentren mit einem erhöhten patientenspezifischen Risikoprofil ihre Ergebnisqualität adäquater einordnen können. Darüber hinaus werden kurzfristige Ergebnisse zu infektionsbedingten Folgeeingriffen sowie zu sogenannten schwerwiegenden Folgeeingriffen dargestellt. Letztere sind definiert als Revisionseingriffe mit Aus- oder Einbau mindestens einer am Knochen verankerten Komponente (Schaft oder Pfanne bzw. Femur- oder Tibiakomponente) und entsprechen damit konzeptionell der in der EndoCert-Zertifizierung vorgenommenen Differenzierung. Ergänzend werden kurz- und mittelfristige Ergebniskennzahlen auf Implantatsystemebene bereitgestellt. Dies ermöglicht eine strukturierte Analyse implantatspezifischer Ergebnisprofile und unterstützt EndoCert-Zentren dabei, potenzielle Auffälligkeiten frühzeitig zu identifizieren und gezielt in qualitätssichernde Maßnahmen zu überführen.

Seit Januar 2026 steht den zertifizierten Zentren zusätzlich eine EndoCert-spezifische Klinikauswertung zur Verfügung. In dieser werden ausschließlich jene Fälle berücksichtigt, die bei der EPRD-Erfassung als EndoCert-Eingriffe gekennzeichnet wurden, vorausgesetzt, die Fallzuordnung erfolgt konsistent. Die Vergleichsgruppe für das Benchmarking umfasst weiterhin alle im EPRD dokumentierten Eingriffe und ist damit bewusst nicht auf EndoCert-Fälle beschränkt.

#### Interpretationsmöglichkeiten der EPRD-Klinikauswertung im Kontext von EndoCert

Die in der aktuellen Klinikauswertung fokussierte Darstellung kurzfristiger Ergebnisse (ca. 30 Monate) erlaubt es EndoCert-Zentren, frühe Abweichungen in Ausfall- und Komplikationswahrscheinlichkeiten zeitnah zu identifizieren. Die differenzierte Auswertung nach Versorgungsformen (z. B. elektive Hüft-TEP mit zementiertem versus zementfreiem Schaft, Hemiendoprothesen, nichtelektive Versorgungen) ermöglicht eine auditrelevante Zuordnung auffälliger Ergebnisse zu spezifischen Behandlungspfaden. Besonders relevant für EndoCert ist die getrennte Betrachtung

- infektionsbedingter Folgeeingriffe und
- schwerwiegender Folgeeingriffe mit Aus- oder Einbau knochenverankerter Komponenten (Schaft oder Pfanne bzw. Femur- oder Tibiakomponente).

Beide Endpunkte sind unmittelbar qualitätsrelevant: Infektionsbedingte Folgeeingriffe stellen zentrale Indikatoren der perioperativen Versorgungsqualität dar, während schwerwiegende Folgeeingriffe konzeptionell der EndoCert-Systematik entsprechen, die zwischen knochenverankerten und nichtknochenverankerten Komponenten differenziert. Entsprechend sind beide Kategorien direkt im Rahmen der Zertifizierung auditrelevant interpretierbar.

Die zusätzliche Darstellung risikoadjustierter kurzfristiger Ergebnisse stellt einen wesentlichen Zugewinn für EPZ/EPZmax mit komplexem Patientenkollektiv dar, wenngleich dem Register nur begrenzte Informationen zu möglichen Risikofaktoren vorliegen. Sie erlaubt eine Bewertung der eigenen Ergebnisqualität unter Berücksichtigung des patientenspezifischen Risikoprofils und schützt vor Fehlinterpretationen rein unadjustierter Kennzahlen.

Die mittelfristigen Auswertungen (bis ca. 5 Jahre) ergänzen die kurzfristige Perspektive um eine Bewertung der Versorgungsergebnisse. Für EndoCert liefern sie Hinweise darauf, ob sich früh identifizierte Auffälligkeiten persistieren, normalisieren oder verstärken. Die implantatspezifische Differenzierung erlaubt zudem eine kritische Reflexion des Implantatportfolios, wie sie im Rahmen der EndoCert-Anforderungen an die Implantatauswahl und das Risikomanagement gefordert wird.

**Abb. 2:**
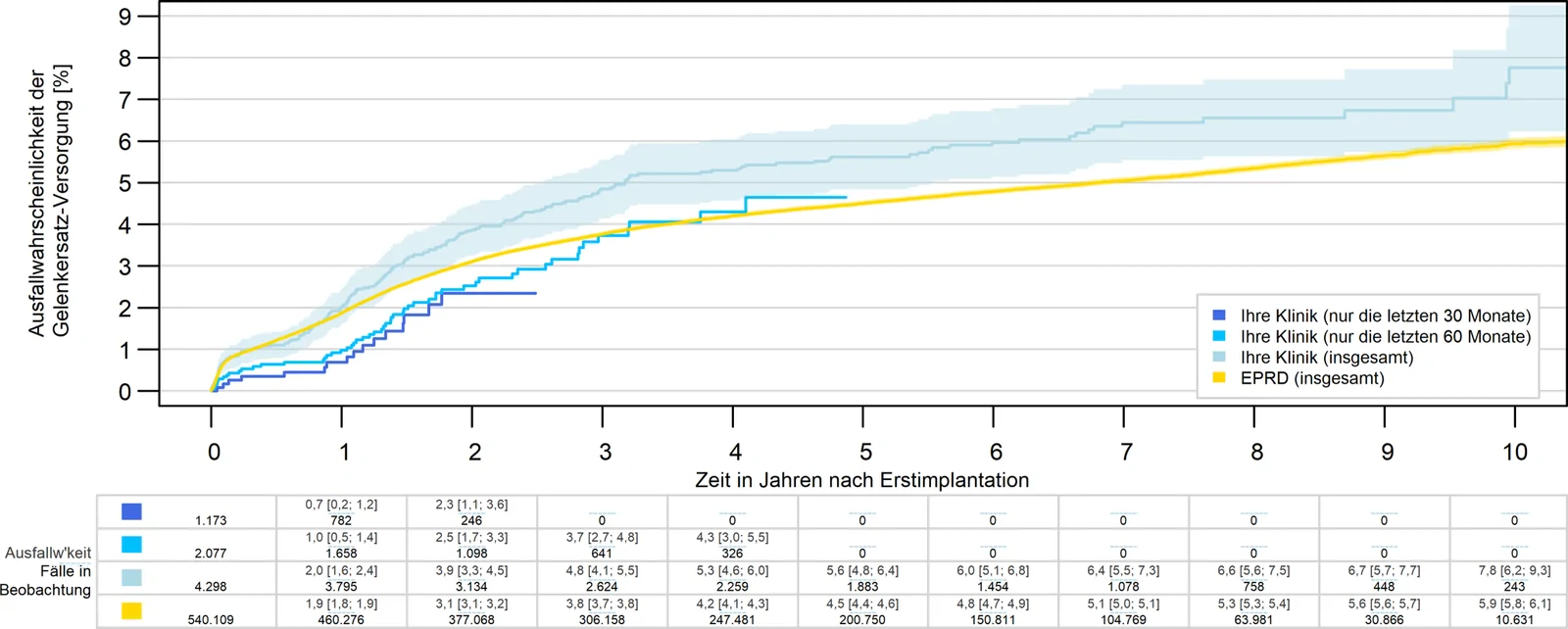
Auszug aus einer EPRD (Endoprothesenregister Deutschland)-Klinikauswertung mit Vergleich zentrenspezifischer und registerweiter Ausfallwahrscheinlichkeiten nach Standardknietotalendoprothese

Die Darstellung der Ausfallwahrscheinlichkeiten im Zeitverlauf (Kaplan-Meier-basierte Analysen) ermöglicht eine dynamische Einordnung der klinikeigenen Ergebnisse im Vergleich zur Gesamtheit der am EPRD teilnehmenden Kliniken (Abb. 2). Für EndoCert ist dies relevant, da Verläufe, Konfidenzintervalle und die Zahl der tatsächlich unter Risiko stehenden Fälle sichtbar werden. Diese Darstellungen fördern eine transparente, datenbasierte Diskussion im Rahmen von Qualitätszirkeln und Komplikationsbesprechungen, wie sie EndoCert explizit fordert.

Ergänzend wurden in einer früheren Arbeit bereits konkrete Interpretationshilfen zur Bewertung der EPRD-Berichte unter Berücksichtigung der EndoCert-Anforderungen veröffentlicht, die sich sowohl an am EPRD teilnehmende Kliniken als auch an EndoCert-Fachexperten richten und die strukturierte Einordnung dieser Auswertungen im Auditprozess unterstützen [10].

Erst seit Januar 2020 ist die Kennzeichnung als EndoCert-Eingriff mit dem sogenannten EndoCert-Button möglich. Hintergrund ist, dass die EPRD-Meldung zumeist zentrumsweit für alle Abteilungen einschließlich Belegärzte erfolgt und nicht automatisch auf die zertifizierten Versorgungsstrukturen begrenzt ist. Die EndoCert-Kennzeichnung ermöglicht daher eine gezielte Differenzierung zertifizierungsrelevanter Eingriffe innerhalb der Gesamtdokumentation. Je nachdem, ab wann eine EndoCert-Klinik die Funktion des EndoCert-Buttons nutzte, ist bei der Interpretation der EndoCert-spezifischen Auswertung zu berücksichtigen, dass folglich darin weniger Erstimplantationen enthalten sind als in der allgemeinen Klinikauswertung, welche alle Eingriffe seit Erstmeldung der jeweils teilnehmenden EPRD-Klinik umfasst. Dieser Umstand ist insbesondere bei der Beurteilung von Ergebnissen über den Gesamtzeitraum seit Registrierung beim Register zu berücksichtigen, während kurz- und mittelfristige Ergebnisdarstellungen hiervon nicht relevant beeinflusst werden, da in diesen Beobachtungszeiträumen bereits alle relevanten EndoCert-Eingriffe auch als solche gekennzeichnet sind.

### PROM-Bericht: Übersicht der Befragungsergebnisse

Die Standzeit einer Endoprothese stellt nur einen Indikator für die Ergebnisqualität dar, die subjektive Beurteilung der Versorgung durch den Patienten selbst einen weiteren.

#### Relevante EndoCert-Anforderungen zur Ergebnisqualität

Im EndoCert-System war die Erhebung patientenberichteter Ergebnismessungen (PROM) seit Beginn vorgesehen, jedoch lediglich fakultativ implementiert und damit weder systematisch noch flächendeckend Bestandteil der externen Qualitätssicherung [7].

#### PROM-Übersicht aus dem EPRD

**Abb. 3:**
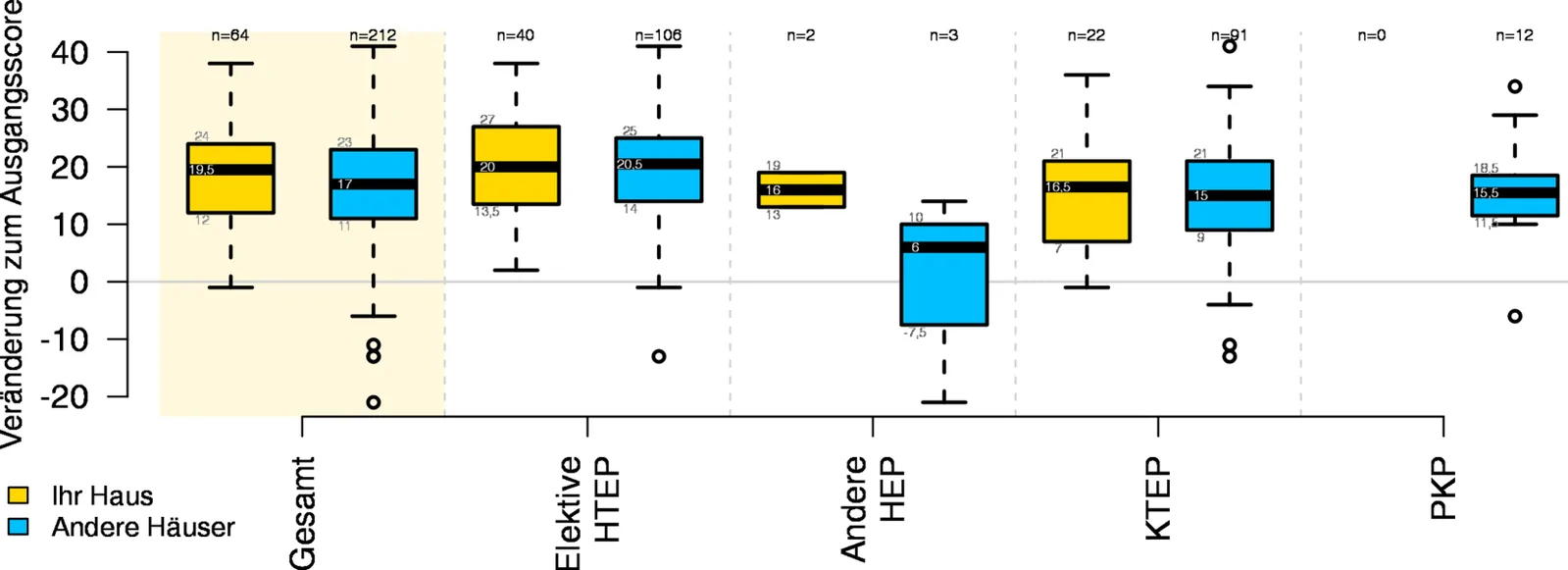
Auszug aus einer EPRD-Muster-PROM-Übersicht. *Elektive HTEP* Alle Hüfttotalendoprothesen, die nicht aufgrund einer Femurfraktur vorgenommen wurden, *HEP* Andere Hüftendoprothesen fasst nicht-elektive HTEP und Hemiendoprothesen zusammen, *PKP* Partiale Knieprothesen; also vor allem unikondyläre Knieversorgungen, aber auch femoropatellare Versorgungen

Die PROM lassen sich nach einer Phase des Probebetriebs seit 01.01.2025 von allen teilnehmenden Kliniken im EPRD erheben. Um den Aufwand für die Klinik möglichst gering zu halten, findet die Befragung dabei vollständig online statt. Nach einer präoperativen Befragung wird der Patient per E‑Mail auch zu postoperativen Befragungen (nach 6 Monaten, 1 und 2 Jahren) eingeladen. Erfragt wird dabei der bei internationalen Registern geläufige Oxford Hip bzw. Knee Score sowie bei den postoperativen Befragungen auch in einer zusätzlichen Frage die allgemeine Zufriedenheit des Patienten mit der Versorgung [8]. Zweimal jährlich erhalten Kliniken, die über das EPRD an der PROM-Befragung teilnehmen, Rückmeldungen zu den PROM-Ergebnissen ihrer Patienten und können beispielsweise differenziert nach Versorgungsform erkennen, wie sich die Scores von prä- zu postoperativ verändert haben (Abb. 3) und wie zufrieden die Patienten mit ihrem Kunstgelenk sind [4].

Die Möglichkeit der PROM-Erfassung über das EPRD verlagert nicht nur erhebliche organisatorische und personelle Aufwände von den Einrichtungen hin zum Register, sondern schafft zugleich die Voraussetzung, um PROM-Daten perspektivisch systematisch in die Interpretation der im Rahmen von EndoCert zu prüfenden Ergebnisqualität einzubeziehen.

#### Interpretationsmöglichkeiten des EPRD-PROM-Berichts im Kontext von EndoCert

Die PROM-Übersichten des EPRD ermöglichen EndoCert-Zentren eine strukturierte Einordnung der patientenberichteten Behandlungsergebnisse ergänzend zu den klinischen Ergebnisparametern [3, 4]. Sie erlauben die Beurteilung, inwieweit funktionelle Verbesserungen, Schmerzreduktion und gesundheitsbezogene Lebensqualität aus Patientensicht erreicht werden und ob diese mit den registerbasierten Ergebniskennzahlen konsistent sind. Darüber hinaus können zeitliche Verläufe sowie Unterschiede zwischen Versorgungsformen oder Patientengruppen identifiziert und in den Qualitätszirkeln reflektiert werden.

Die Ergebnisse der im Rahmen der postoperativen Patientenbefragung erhobenen Patientenzufriedenheit können zudem genutzt werden, um für die EndoCert-Zertifizierung die Zufriedenheit der Patientinnen und Patienten mit dem unmittelbaren Versorgungsprozess zu beurteilen.

### Jahresbericht

Obwohl der EPRD-Jahresbericht nicht Bestandteil der auditrelevanten EndoCert-Auswertung ist, bietet dieser eine übergeordnete, nationale Referenz zur endoprothetischen Versorgung in Deutschland und stellt damit Trends und Versorgungsrealitäten dar, die über den zentrenspezifischen Fokus der Klinikauswertungen hinausgehen. Im Rahmen der EndoCert-Zertifizierung sollte der Jahresbericht daher vor allem als Hintergrundquelle genutzt werden, um strukturelle Entwicklungen, etwa Veränderungen der Versorgungsformen, Implantatverwendung oder Fallzahlen, im nationalen Kontext zu verstehen. Zudem liefert der Jahresbericht Hinweise auf Implantatsysteme mit hohem Revisionsrisiko und sollte deshalb dem verantwortungsvollen Operateur bekannt sein, um gegebenenfalls die Implantatwahl umzustellen. Eine direkte Bewertung der zentrenspezifischen Ergebnisqualität erfolgt jedoch ausschließlich über die EPRD-Klinikauswertung [10].

## Synthese und Ausblick: EPRD-Daten als Steuerungsinstrument der EndoCert-Zertifizierung

Obwohl EndoCert und das EPRD komplementäre Ziele verfolgen, die Sicherstellung hoher Versorgungsqualität einerseits und die umfassende Abbildung endoprothetischer Versorgungsergebnisse andererseits, erfolgte die Verzahnung beider Systeme lange Zeit nicht systematisch. Registerdaten wurden in den frühen Auditphasen bereits berücksichtigt, jedoch fehlten lange Zeit standardisierte Auswertungskriterien, definierte Referenzwerte sowie ein einheitlicher Bewertungsrahmen, der eine auditrelevante und reproduzierbare Interpretation der Registerinformationen ermöglicht hätte. Zusätzlich war im EPRD über viele Jahre nicht ersichtlich, welche Eingriffe unter EndoCert-Bedingungen durchgeführt wurden. Diese Lücke wurde erst durch die Einführung einer expliziten Kennzeichnung sogenannter EndoCert-Fälle geschlossen und schafft seit 2026 die Grundlage für eine deutlich belastbarere, zentrenspezifische Auswertung der Registerdaten.

Die vorliegende Übersicht zeigt, dass die im EPRD verfügbaren Daten ein zentrales Instrument zur Bewertung und Weiterentwicklung der Ergebnisqualität im Rahmen der EndoCert-Zertifizierung darstellen. Insbesondere die zentrenspezifischen Auswertungen ermöglichen es zertifizierten Einrichtungen, ihre Versorgungsrealität differenziert abzubilden, qualitätsrelevante Auffälligkeiten frühzeitig zu erkennen und diese strukturiert in interne Qualitäts- und Risikomanagementprozesse zu überführen.

Deutlich wird, dass der Nutzen von Registerdaten nicht in der bloßen Darstellung von Ergebnissen liegt, sondern in ihrer methodisch fundierten und kontextualisierten Interpretation unter Einbeziehung von Risikoprofil, Versorgungsform und Implantatstrategie [10]. Vor dem Hintergrund internationaler Registererfahrungen, die auch in der Hüftendoprothetik eine Abnahme von Revisionswahrscheinlichkeiten belegen [1, 9], ist eine konsequente, strukturierte Auswertung der EPRD-Daten durch EndoCert-Zentren erforderlich, um vergleichbare Qualitätsentwicklungen auch im nationalen Versorgungssystem zu ermöglichen.

Perspektivisch bieten die fortlaufenden Weiterentwicklungen der EPRD-Klinikauswertung, speziell die stärkere Fokussierung auf kurzfristige und risikoadjustierte Ergebnisse sowie fallbezogene Analysen, die Grundlage für eine noch engere Verzahnung von Registerdaten, Zertifizierungsanforderungen und klinischer Qualitätssteuerung. Mit der seit 2025 etablierten Möglichkeit, patientenberichtete Ergebnismessungen strukturiert über das EPRD zu erheben, erweitert sich dieser Ansatz erstmals systematisch um die Perspektive der Patientinnen und Patienten. Damit können klinische Ergebniskennzahlen künftig um patientenzentrierte Outcome-Dimensionen ergänzt und differenzierter interpretiert werden. In der Kombination aus objektiven Registerdaten und subjektivem Behandlungserleben liegt ein wesentliches Potenzial, die EndoCert-Zertifizierung über strukturelle und prozessuale Anforderungen hinaus zunehmend als daten- und patientenzentriertes Instrument zur nachhaltigen Verbesserung der endoprothetischen Versorgung in Deutschland zu etablieren.

## Limitation

Die Nutzung der EPRD-Daten als ergänzendes Instrument im Kontext der EndoCert-Zertifizierung bietet wertvolle Einblicke in die Ergebnisqualität, ist jedoch nicht vollständig repräsentativ für alle Aspekte der Versorgung. Unterschiede in der Meldequote oder unbeantwortete Rückfragen zu einzelnen Fällen können zu einer teilweisen Ausschließung von Daten führen. Zudem spiegeln EPRD-Daten nur die im Register dokumentierten Fälle wider, sie liefern daher eine belastbare, aber nicht vollständige Abbildung der klinischen Versorgungsrealität.

Eine darüberhinausgehende Einschränkung ergibt sich aus registerinhärenten methodischen Unterschieden zwischen den teilnehmenden Zentren, etwa in der Kodierpraxis, der Vollständigkeit der Nachverfolgung sowie der zeitlichen Tiefe der verfügbaren Verlaufsdaten. Diese Faktoren können die Vergleichbarkeit einzelner Kennzahlen beeinflussen und sind bei der Interpretation zentrenspezifischer Ergebnisse zu berücksichtigen.

## Fazit für die Praxis

- Die EPRD(Endoprothesenregister Deutschland)-Klinikauswertung stellt für EndoCert-Zentren das zentrale Instrument zur strukturierten Bewertung der Ergebnisqualität dar.
- Eine valide Interpretation der Registerdaten setzt eine hohe Meldequote sowie die Berücksichtigung von Versorgungsform, Risikoprofil und Implantatstrategie voraus.
- Kurz- und mittelfristige Auswertungen ermöglichen es, früh Auffälligkeiten zu erkennen und gezielt in das klinische Risiko- und Qualitätsmanagement einzubinden.
- Risikoadjustierte Ergebnisdarstellungen unterstützen insbesondere Zentren mit komplexem Patientenkollektiv bei der Bewertung ihrer Versorgungsqualität.
- Implantatspezifische Analysen aus der Klinikauswertung fördern die Weiterentwicklung des Implantatportfolios.
- Die fallbezogenen Zusatzdaten (Einzelfalllisten) ermöglichen eine tiefere Ursachenanalyse.
- Die strukturierte Nutzung der EPRD-Daten im EndoCert-Kontext stärkt Komplikationsbesprechungen, Qualitätszirkel und Leitungsgremien als zentrale Steuerungsinstrumente.

## Data Availability

Die Daten sind auf begründete Anfrage beim korrespondierenden Autor erhältlich.

## Abkürzungen

ASA: American Society of Anesthesiologists
BMI: Body-Mass-Index
EPRD: Endoprothesenregister Deutschland
EPZ: Endoprothetikzentrum
EPZmax: Endoprothetikzentrum der Maximalversorgung
PROM: „Patient-reported outcome measures“
TEP: Totalendoprothese
